# TH1 cell-inducing *Escherichia coli* strain identified from the small intestinal mucosa of patients with Crohn’s disease

**DOI:** 10.1080/19490976.2020.1788898

**Published:** 2020-07-20

**Authors:** Manabu Nagayama, Tomonori Yano, Koji Atarashi, Takeshi Tanoue, Mariko Sekiya, Yasutoshi Kobayashi, Hirotsugu Sakamoto, Kouichi Miura, Keijiro Sunada, Takaaki Kawaguchi, Satoru Morita, Kayoko Sugita, Seiko Narushima, Nicolas Barnich, Jun Isayama, Yuko Kiridooshi, Atsushi Shiota, Wataru Suda, Masahira Hattori, Hironori Yamamoto, Kenya Honda

**Affiliations:** a Department of Medicine, Division of Gastroenterology, Jichi Medical University, Tochigi, Japan; b Department of Microbiology and Immunology, Keio University School of Medicine, Tokyo, Japan; c Laboratory for Gut Homeostasis, RIKEN Center for Integrative Medical Sciences, Yokohama, Japan; d JSR-Keio University Medical and Chemical Innovation Center, Tokyo, Japan; e UMR 1071 Inserm/Université Clermont Auvergne; USC-INRA 2018, Microbes, Intestin, Inflammation et Susceptibilité de l’Hôte (M2iSH), CRNH Auvergne, Clermont-Ferrand, France; f Cooperative Major in Advanced Health Science, Graduate School of Advanced Science and Engineering, Waseda University, Tokyo, Japan

**Keywords:** Crohn’s disease, double-balloon enteroscopy, microbiome, *Escherichia coli*, *Ruminococcus gnavus*, T_H_1, T_H_17

## Abstract

Dysbiotic microbiota contributes to the pathogenesis of Crohn’s disease (CD) by regulating the immune system. Although pro-inflammatory microbes are probably enriched in the small intestinal (SI) mucosa, most studies have focused on fecal microbiota. This study aimed to examine jejunal and ileal mucosal specimens from patients with CD via double-balloon enteroscopy. Comparative microbiome analysis revealed that the microbiota composition of CD SI mucosa differs from that of non-CD controls, with an increased population of several families, including Enterobacteriaceae, Ruminococcaceae, and Bacteroidaceae. Upon anaerobic culturing of the CD SI mucosa, 80 bacterial strains were isolated, from which 9 strains representing 9 distinct species (*Escherichia coli, Ruminococcus gnavus, Klebsiella pneumoniae, Erysipelatoclostridium ramosum, Bacteroides dorei, B. fragilis, B. uniformis, Parabacteroides distasonis*, and *Streptococcus pasteurianus*) were selected on the basis of their significant association with CD. The colonization of germ-free (GF) mice with the 9 strains enhanced the accumulation of T_H_1 cells and, to a lesser extent, T_H_17 cells in the intestine, among which an *E. coli* strain displayed high potential to induce T_H_1 cells and intestinal inflammation in a strain-specific manner. The present results indicate that the CD SI mucosa harbors unique pro-inflammatory microbiota, including T_H_1 cell-inducing *E. coli*, which could be a potential therapeutic target.

## Introduction

Crohn’s disease (CD) is a debilitating chronic gastrointestinal disease primarily affecting the ileum and colon and is characterized by longitudinal ulcers, aphthae, and stenosis, which are distinct from the characteristics of ulcerative colitis. Genome-wide association studies have revealed more than 100 CD-associated genes, including those encoding NOD2, IL23R, ATG16L1, and TNFSF15.^1-3^ In addition to host intrinsic factors, extrinsic factors including the gut microbiota are potentially involved in the pathogenesis of CD. Indeed, the number of CD patients has drastically increased in countries including Japan, probably owing to changes in diet and lifestyle.

The gut microbiota is a potent regulator of host immunity and is reportedly associated with several diseases.^4,5^ Increasing evidence indicates that alteration of the microbiota (called microbiota dysbiosis) affects CD pathogenesis. For example, a reduction of the α-diversity of the microbiota and a reduced abundance of *Faecalibacterium prausnitzii* have been frequently reported in the feces of patients with CD.^6^ Moreover, although numerous studies have focused on the fecal microbiota, limited information is available regarding small intestinal (SI) mucosal microbiota, which is highly likely to influence CD pathogenesis.

The double-balloon endoscopy (DBE) system comprises a fiberscope and a balloon-pump system, which enables more detailed inspection of the SI (distal jejunum and proximal ileum) in comparison with conventional push enteroscopy.^7-9^ This study aimed to examine jejunal and ileal mucosal specimens from patients with CD via DBE. We collected SI mucosal specimens from CD patients and non-CD controls through biopsy or scraping using DBE. The mucosal specimens were subjected to 16S ribosomal RNA (rRNA) gene sequencing analysis and bacterial isolation. By combining the gnotobiotic technique with anaerobic culturing of the mucosal microbiota, we identified an *Escherichia coli* strain with a strong capability to promote T_H_1 cell accumulation in the intestine when colonized in germ-free (GF) mice.

## Results

### Unique microbiota structure in the SI mucosa of CD patients

**Figure 1.**
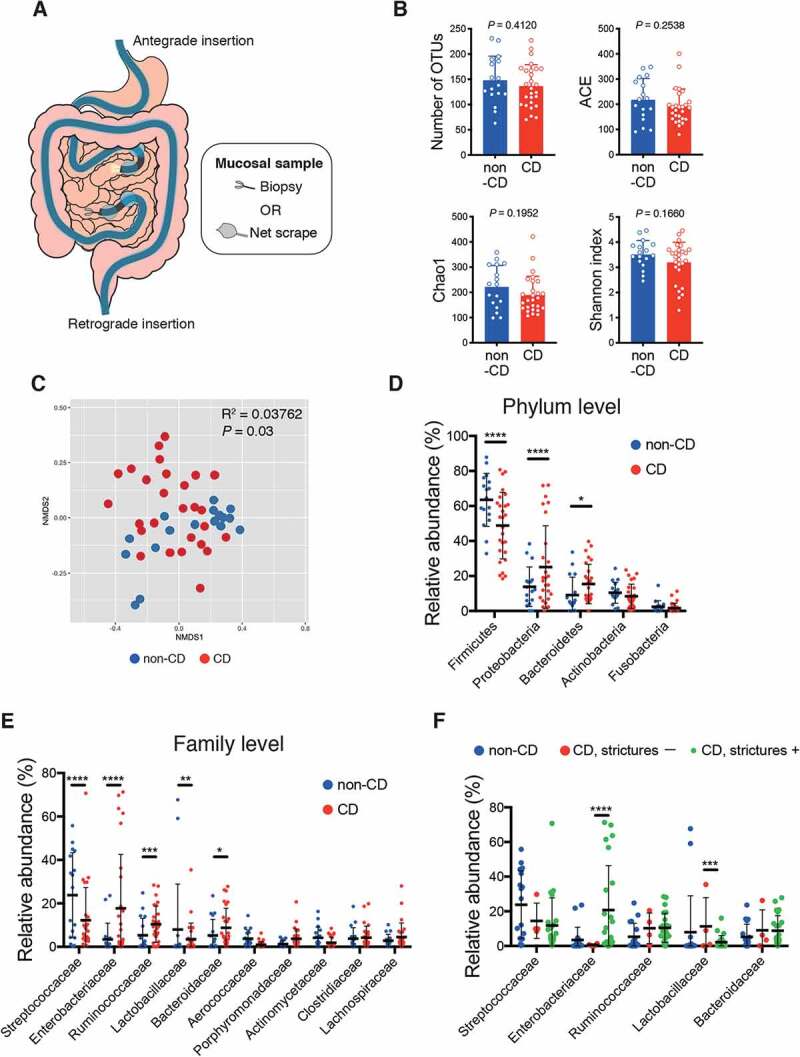
Unique microbiota structure in the small intestinal (SI) mucosa of Crohn’s disease (CD) patients. (a) Schematic representation of specimens obtained from the SI via double-balloon enteroscopy. 16S rRNA gene sequencing analyses were conducted (CD; n = 27, non-CD; n = 17). (b) Alpha-diversity indices of the SI microbiota. (c) Bray-Curtis distance-based non-metric multidimensional scaling (NMDS) plot of SI samples and permutational multivariate analysis of variance (PERMANOVA) statistics between CD (red) and non-CD samples (blue). (d-f) Relative abundance of bacterial taxa at the phylum (d) and the family level (e, f) of the SI microbiota. (f) Statistical comparison between CD patients with (green) and without (red) intestinal stricture(s). Each bar in (B) and the thick bar in (D-F) represent the mean values of groups. Each point in (b-f) represents an individual sample. Error bar, SD. *P* or FDR value * < 0.05; ** < 0.01; *** < 0.001; **** < 0.0001, unpaired Student’s t-test (B), multiple t-tests with false discovery rate (FDR) approach (D-F).

SI mucosal specimens were obtained from 27 CD and 17 non-CD patients (with extra-SI disease, hamartomatous disease, etc., see Table 1) through biopsy or scraping during the DBE method (Figure 1a**, Fig. S1A, B**). Among CD patients, 23 patients had intestinal stricture(s) (Montreal classification B2 or B3), and 19 received treatment with anti-tumor necrosis factor-α (TNF-α) antibody (Table 1). The insertion route of DBE and the sampling method were determined by the endoscopist on the basis of the patients’ condition. When the antegrade (per-oral) insertion route was selected, the distal jejunal mucosa was sampled; retrograde (per-anal) insertion, proximal ileal mucosa. SI specimens were obtained from the mucosa outside of active ulcers or strictures. We conducted 16S rRNA gene sequencing analyses using the Illumina MiSeq platform, and 2,660 operational taxonomic units (OTUs) were detected from the 44 SI mucosal samples. Although the α-diversity did not significantly differ (Figure 1b), the microbiota composition significantly differed between CD patients and non-CD controls, as revealed through the Bray-Curtis distance-based non-metric multidimensional scaling (NMDS) plot (Figure 1c). There was no significant difference in the microbiota diversity between CD and non-CD specimens irrespective of insertion route or sampling procedure (**Fig. S2A, F**). SI mucosal microbiota of CD patients contained more taxa within Proteobacteria and Bacteroidetes at the phylum level, and more taxa within Enterobacteriaceae, Ruminococcaceae, and Bacteroidaceae at the family level, as compared to non-CD controls (Figure 1d,e). In contrast, taxa within the Firmicutes phylum and the Streptococcaceae family were reduced in CD patients (Figure 1d,e).

**Table 1.** Demographic characteristics of the patients.

	Patients with CD (n = 27)	Non-CD patients (n = 17)	*P* value
Age (y)	42.6 (±10.5)	51.1 (±17.5)	0.0503
Sex (female, %)	14.8	64.7	0.0011
Route (antegrade/retrograde)	4/23	12/5	0.0003
Sample (biopsy/scrape)	14/13	8/9	> 0.9999
Disease duration (y)	14.1 (±10.6)		
Montreal classification (%)			
L1/L2/L3	74.1/0/25.9		
B1/B2/B3	14.8/81.5/3.7		
Past surgery (%)	33.3		
WBC (/μL)	5,826 (±2,057)	4,694 (±1,070)	0.0426
RBC (×10^4^/μL)	454.7 (±52.3)	436.5 (±68.8)	0.3248
Hb (g/dL)	13.5 (±1.4)	13.1 (±2.3)	0.4251
CRP (mg/dL)	0.3 (±0.5)	0.0 (±0.1)	0.1536
Treatment (%)			
5-ASA	88.9		
Anti-TNF-α antibody	70.4		
Immunomodulators	40.7		
Steroids	3.7		
Elemental diet	81.5		
Probiotics	55.6		
Background disease of non-CD patients		Disease other than SI (n = 6) Peutz-Jeghers syndrome (n = 4) History of intestinal obstruction (n = 3) Gastrointestinal stromal tumor (n = 2) Malignant lymphoma (n = 2) Obscure gastrointestinal bleeding (n = 2) Familial adenomatous polyposis (n = 1) Ischemic enteritis (n = 1)	

Data are presented mean (± SD). Abbreviation; CD = Crohn’s disease, WBC = white blood cells, RBC = red blood cells, Hb = hemoglobin, CRP = C-reactive protein, 5-ASA = 5-aminosalicylic acid. Unpaired Student’s t-test and Fisher’s exact test were used for numerical data and categorical data, respectively.

### E. coli and Ruminococcus gnavus were enriched in the CD SI mucosa

**Figure 2.**
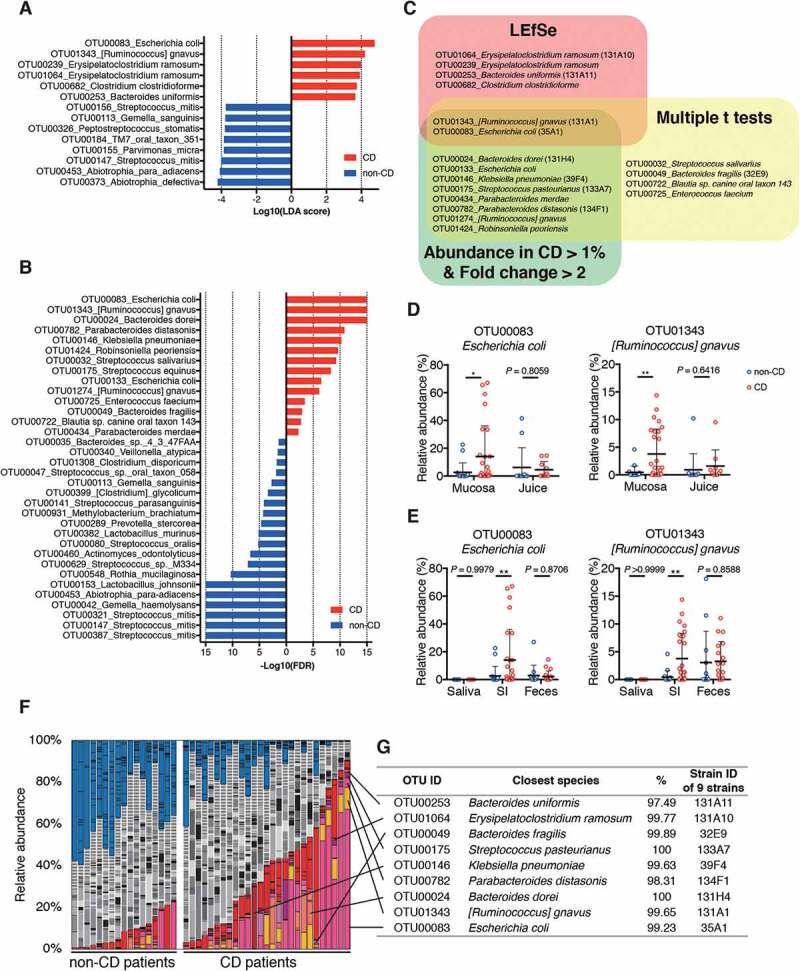
Taxa that were enriched in the small intestinal (SI) mucosa of Crohn’s disease (CD) patients. (a) A histogram of the LDA scores computed for differentially abundant taxa between CD and non-CD subjects. (b) A histogram of the false discovery rate (FDR) calculated by comparing SI samples between CD and non-CD. Only significant taxa (FDR < 0.05) are shown. (c) Venn diagram of 18 CD-enriched bacteria identified via three computational analyses. (d) Relative abundance of *E. coli* and *R. gnavus* in mucosal specimens and intestinal juice. (e) Relative abundance of *E. coli* and *R. gnavus* in the saliva, SI, and feces. (f) SI microbiota composition of each sample was determined via 16S rRNA sequencing. Operational taxonomic units (OTUs) significantly enriched in CD patients are marked in warm colors, those enriched in non-CD controls are in blue, and those without significant differences are indicated in gray. (g) A list of the 9 strains isolated from CD SI samples. Their closest species and percentage similarity (%) in the National Center for Biotechnology Information (NCBI) database are indicated. Each thick bar in (d, e) represents the mean of a group. Each point in (D, E) represents an individual sample. Error bar, SD. *P < .05; **P < .01, LEfSe (A), multiple t-tests with FDR approach (B), unpaired Student’s t-test (D, E).

To identify differential microbial taxa between CD and non-CD patients, we used the following two comparison-based statistical methods: linear discriminate analysis (LDA) coupled with effect size measurements (LEfSe) and multiple t-tests with the false discovery rate (FDR) approach. LEfSe revealed 6 OTUs with a significantly higher abundance in the SI mucosa of CD patients (Figure 2a), whereas multiple t-tests identified 14 OTUs associated with CD (Figure 2b). Furthermore, we mined the sequence data for OTUs exhibiting high abundance in the CD-associated SI mucosa [mean abundance in the SI mucosa of CD patients > 1% and fold change (CD/non-CD) > 2], and 10 OTUs met these criteria (Figure 2c, Table S1). The combination of these three computational analyses identified 18 CD-associated OTUs (Figure 2c, Table S1). OTUs sharing identity with *E. coli, Ruminococcus gnavus, Bacteroides dorei, Klebsiella pneumoniae, Streptococcus pasteurianus, Parabacteroides merdae, Parabacteroides distasonis, and Robinsoniella peoriensis* were significantly associated with CD in at least two analyses, among which *E. coli* and *R. gnavus* were significantly associated with the CD SI mucosa in all three analyses in terms of abundance (Figure 2c). *E. coli* and *R. gnavus* were enriched in CD specimens irrespective of insertion route or sampling procedure (**Fig. S2C-E, H-J**). In addition, these OTUs sharing identity with *E. coli* and *R. gnavus* were more prevalent in CD patients (detected in more than 70% patients with >0.1% abundance) than in controls (Table S1**, Fig. S3A**). Additionally, SI mucosal microbiota of CD patients with intestinal strictures or treated with anti-TNF-α antibody therapy contained more taxa within Enterobacteriaceae as compared to that of the other CD patients (Figure 1f**, Fig. S3B**).

We also compared the microbiota of CD and non-CD SI mucosa with those of saliva and feces collected one day before the DBE examination. The Bray-Curtis distance-based NMDS plot showed that the microbiome of SI mucosa, saliva, and feces from CD or non-CD patients were distinct from each other (**Fig. S3C**). Concurrent with previous reports,^10,11^ the fecal microbiota of CD patients had a reduced α-diversity (**Fig. S3D**) and a reduced abundance of *Faecalibacterium prausnitzii*, as compared with non-CD controls (**Fig. S3E, F)**. Distinct from the fecal microbiota, the α-diversity (Figure 1b) and the abundance of *F. prausnitzii* (**Fig. S3F)** in the SI mucosa were similar between the groups. Importantly, a markedly stronger association between *E. coli, R. gnavus*, and *K. pneumoniae* with CD was observed for the SI mucosa than the fecal microbiota (Figure 2e**, S4A-C**). We also examined the SI juice aspirated before collection of mucosal specimens and found that OTUs related to *E. coli* and *R. gnavus* were less abundant in the juice as compared with mucosal samples (Figure 2d). These results suggest that *E. coli* and *R. gnavus* were present in the SI mucus layer or adhered to the epithelial cells. Although *E. coli* and *R. gnavus* have been previously associated with CD in large studies that profiled the fecal microbiome,^12,13^ our results suggested that analysis of the SI mucosa microbiome is more powerful than that of the fecal microbiome, and a smaller sample size would be sufficient for detection of potentially pathogenic bacteria in the context of CD.

### Induction of intestinal T_H_1 cells by the CD-associated E. coli strain

SI mucosal samples of CD patients were further subjected to aerobic and anaerobic culturing using several media, and 80 bacterial strains were isolated with distinct 16S rRNA gene sequences (with >98% sequence identity) (**Fig. S5A, B)**. From the 80 isolates, based on their significant association with CD via the aforementioned statistical analyses (multiple t-tests and/or LEfSe, Figure 2c), 9 strains with >97% 16S rRNA gene sequence similarity with *E. coli* (strain ID: 35A1), *R. gnavus* (strain ID: 131A1), *B. dorei* (strain ID: 131H4), *K. pneumoniae* (strain ID: 39F4), *S. pasteurianus* (strain ID: 133A7), *P. distasonis* (strain ID: 134F1), *Bacteroides fragilis* (strain ID: 32E9), *Erysipelatoclostridium ramosum* (strain ID: 131A10), and *Bacteroides uniformis* (strain ID: 131A11) (figure 2f**, G, Table S1**) were selected for follow-up analysis. The identity of *E. coli* 35A1 strain as *E. coli* was further confirmed through a biochemical examination using several diagnostic identification media and the API 20E system (**Fig. S6**).

**Figure 3.**
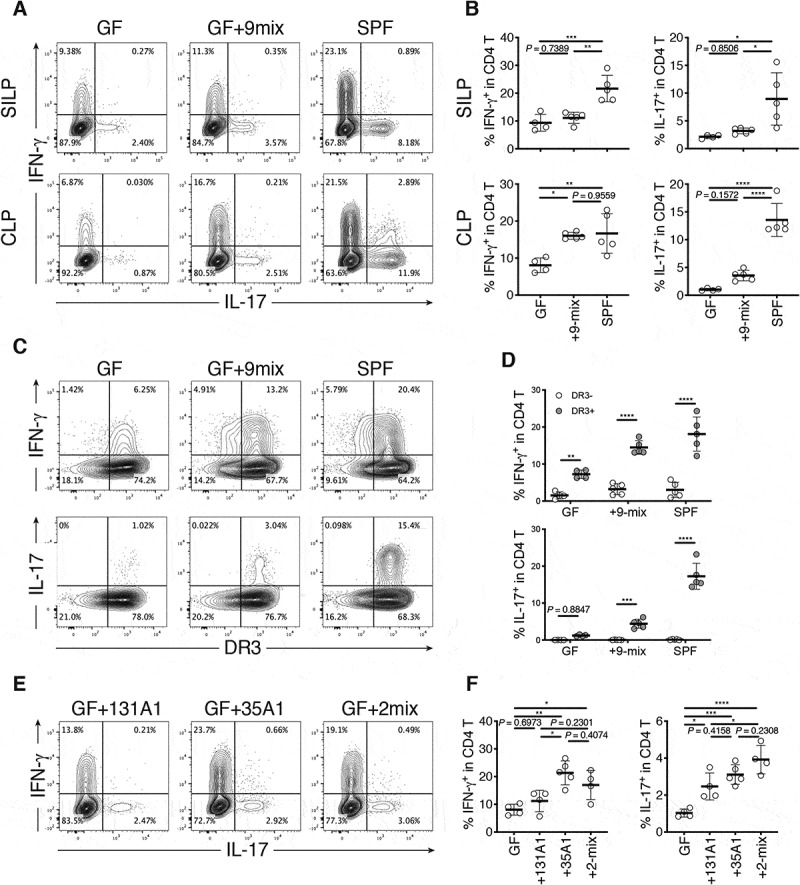
Induction of intestinal T_H_1 cells by Crohn’s disease (CD)-associated bacteria and the *E. coli* strain. (a) Representative flow cytometry plots of small intestinal (upper) and colonic (lower) lamina propria (SILP and CLP) CD4^+^ TCRβ^+^ T cells (CD4 T cells). Germ-free (GF) C57BL/6 (B6) mice were orally inoculated with a mixture of 9 CD-enriched strains (9-mix) and euthanized after 3 weeks. (b) Percentage of IFN-γ^+^ and IL-17^+^ cells among small intestinal (upper) and colonic (lower) LP CD4 T cells. (c) Representative flow cytometry plots of colonic LP CD4 T cells. (d) Percentage of IFN-γ^+^ and IL-17^+^ cells among DR3-positive and DR3-negative CD4 T cells. (e) Representative flow cytometry plots of colonic LP CD4 T cells. GF B6 mice were orally inoculated with a mixture of CD-derived *R. gnavus* and *E. coli* strains (2-mix) or with either of them individually. (f) Percentage of IFN-γ^+^ and IL-17^+^ cells among colonic LP CD4 T cells. Each point in (B, D, F) represents an individual mouse. Each thick bar in (B, D, F) represents the mean of a group. Error bar, SD. *P < .05; **P < .01; ***P < .001; ****P < .0001, one-way ANOVA with Tukey’s post hoc test (b, f), two-way ANOVA with Bonferroni’s post hoc test (D).

As the gut microbiota has been recognized as a potent regulator of the host immune system,^14^ we further analyzed the selected 9 strains for their ability to stimulate immune cells *in vivo* using GF mice. Accordingly, we cultured each of the 9 strains individually and mixed them together to generate a bacterial mixture (9-mix), which was then orally administered to GF C57BL/6 (B6) mice via oral gavage (Figure 2g). The mice were housed in gnotobiotic vinyl isolators for 3 weeks, and lymphocytes in the SI and colonic lamina propria (LP) were examined by flow cytometry (**Fig. S7**). Although the effect on the SI lymphocytes was weak (see Discussion), marked accumulation of interferon-γ^+^ (IFN-γ^+^) CD4T cells [T helper 1 (T_H_1) cells] was observed in the colonic LP of mice colonized with 9-mix, and the level was similar to that of specific pathogen-free (SPF) mice (Figure 3a,b). Although lower than that of T_H_1 cells, the frequency of interleukin-17^+^ (IL-17^+^) CD4T cells [T helper 17 (T_H_17) cells] noticeably increased upon colonization with the 9-mix (Figure 3b). The induced T_H_1 and T_H_17 cells expressed death receptor 3 (DR3) (Figure 3c,d), a receptor of TNFSF15 implicated in CD pathogenesis.^1,15^

Among the 9-mix, we examined the mixture of *E. coli* 35A1 and *R. gnavus* 131A1 (2-mix) or either of them individually (Figure 3e,f), as these two stains were both most abundant and prevalent in the CD mucosa (Figure 2, **Table S1**). Colonization of GF B6 mice with the 2-mix or the *E. coli* 35A1 strain induced T_H_1 cell accumulation in the intestine at the same level as that induced by the 9-mix, whereas the T_H_1 induction effect by *R. gnavus* 131A1 was marginal (Figure 3e,f). *E. coli* 35A1 mainly colonized the colon and cecum (**Fig. S8A**), reflecting greater T_H_1 cell induction in the colon compared with the small intestine. We also compared the effect of colonization with *E. coli* 35A1 and 8-mix (9-mix without *E. coli*). *E. coli* 35A1 monocolonization resulted in significantly higher T_H_1 cell accumulation as compared to 8-mix colonization (**Fig. S8B**), suggesting that *E. coli* 35A1 was the major contributor to the observed accumulation of T_H_1 cells induced by the 9-mix. In addition to T_H_1 cells, the 2-mix and *E. coli* 35A1 induced a significant increase in the frequency of T_H_17 cells to a level similar to that induced by the 9-mix, but not to the level in SPF mice (compare Figure 3b,f).

**Figure 4.**
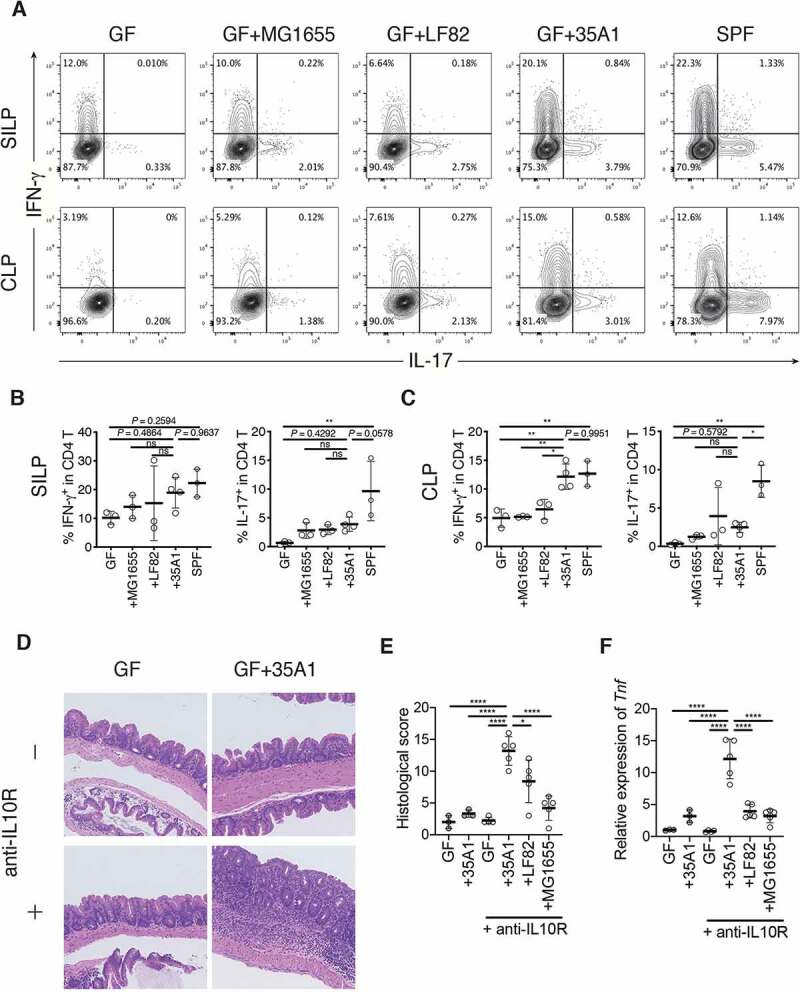
Strain-dependent induction of T_H_1 cells and intestinal inflammation by *E. coli* 35A1. (a) Representative flow cytometry plots of small intestinal (upper) and colonic (lower) lamina propria (SILP and CLP) CD4 T cells. Germ-free (GF) C57BL/6 (B6) mice were orally inoculated with CD-derived *E. coli* 35A1, *E. coli* LF82, or *E. coli* MG1655 strain and euthanized after 3 weeks. (b, c) Percentage of IFN-γ^+^ and IL-17^+^ cells among SI (b) and colonic (c) LP CD4 T cells. (d-f) Representative hematoxylin and eosin staining (d), histological colitis scores of the cecum (e), and relative expression of *Tnf* mRNA in the scraped colonic epithelium (f) of *E. coli* 35A1-, LF82-, or MG1655-monocolonized colitis model mice. GF wild-type B6 mice were colonized with each *E. coli* strain on day 1, followed by weekly intraperitoneal injections with anti-mouse IL10 R antibody (1 mg/body) from day 1 until the end of the experiment. Each point in (B, C, E, F) represents an individual mouse. Each thick bar in (B, C, E, F) represents the mean of a group. Error bar, SD. *P < .05; **P < .01; ns, not significant (*P* > .05), one-way ANOVA with Tukey’s post hoc test.

We then investigated whether the effects of *E. coli* 35A1 on T_H_1 and T_H_17 cells were strain-specific or species-specific. Hence, we generated mice monocolonized with *E. coli* LF82, an adherent-invasive *E. coli* strain previously isolated from a patient with CD,^16^ or with *E. coli* MG1655, a nonpathogenic laboratory strain derived from *E. coli* K-12. In contrast to *E. coli* 35A1, *E. coli* LF82 and *E. coli* MG1655 were less capable of inducing T_H_1 cells in the colonic LP, although marginal effects by *E. coli* LF82 were observed among T_H_1 and T_H_17 cells in the SI and colon (Figure 4a-c). These results suggested that intestinal T_H_1 cells can be induced by *E. coli* in a strain-specific manner.

To examine the causative role of the T_H_1 cell-inducing *E. coli* 35A1 strain in pathogenesis, we employed the anti-IL10 receptor (IL10R) antibody-mediated colitis model.^17^ GF wild-type B6 mice were monocolonized with the *E. coli* 35A1, LF82, or MG1655 strain on day 1, followed by weekly intraperitoneal injections with anti-IL10R antibody. Colonization with the *E. coli* 35A1 strain, but not with the other strains, induced strong intestinal inflammation in the cecum and proximal colon and upregulation of TNF-α in the colonic epithelial cell fraction (Figure 4d-f, **Fig. S8C-F**). These results highlighted the strain-specific pro-inflammatory characteristics of *E. coli* associated with the CD SI mucosa.

## Discussion

CD has been associated with gut microbiota dysbiosis, characterized by reduced α-diversity and an increased population of potentially pro-inflammatory bacteria.^11^ However, only a few bacterial strains have been isolated from CD-associated microbiota and shown to activate the host immune system. Herein, we assessed the SI microbiota of CD patients and screened for immunostimulatory bacteria. We obtained SI samples from patients with CD using the DBE method, which allows for thorough inspection and bacterial isolation from distal and proximal SI unlike standard ileo-colonoscopy. We generated a culture collection comprising 80 bacterial isolates and found that one of the isolates, *E. coli* 35A1, strongly induced intestinal T_H_1 cells.

Although isolated from the SI mucosa, *E. coli* 35A1 mainly colonized the colon, corresponding to greater T_H_1 cell induction in the colon than in the SI. Considering the differences in the intestinal environment between humans and mice, it is perhaps not surprising that human SI-derived isolates exert their effects in a different anatomical site in mice. Although further investigation is required to establish the role of *E. coli* in the pathogenesis of human CD, our data suggest that CD SI mucosa-associated *E. coli* may have substantial pro-inflammatory properties. In this context, *E. coli* LF82 is a clinically relevant strain previously isolated from a CD patient. *E. coli* LF82 can adhere to and invade intestinal epithelial cells (ECs) and replicate within macrophages; accordingly, it has been called adherent-invasive *E. coli* (AIEC).^16,18,19^ Several virulence mechanisms of AIEC have been described, including mucin degradation (Vat-AIEC protease), type 1 pili-mediated adhesion to CEACAM6 (expressed on the apical surface of ECs), and flagella-mediated toll-like receptor (TLR) 5 activation.^20-24^ The 35A1 strain represents another example of inflammatory *E. coli* that was associated with SI mucosa of a patient with CD. Interestingly, *E. coli* 35A1 was more potent than *E. coli* LF82 in inducing T_H_1 cells. The mechanism underlying *E. coli* 35A1-mediated T_H_1 cell induction warrants future investigation. Considering that *Bifidobacterium longum* and *K. pneumoniae* strains have also been associated with intestinal T_H_1 cell induction,^25,26^ *E. coli* 35A1 may have factors similar to those possessed by *B. longum* and *K. pneumoniae*.

In our analysis of SI mucosal microbiota, *R. gnavus* was also found to be associated with CD. *R. gnavus* is reportedly increased in CD, thereby being implicated in its pathogenesis.^27^ *R. gnavus* comprises at least two clades, one of which is enriched in CD patients and produces inflammatory polysaccharides, which induce the production of TNF-α by dendritic cells via TLR4.^28,29^ Although the immunostimulatory effects of *R. gnavus* 131A1 isolated herein were weak, *R. gnavus* may facilitate the colonization of other bacteria associated with CD pathogenesis. Indeed, *R. gnavus* is a mucolytic bacterium and degrades the mucus layer, thereby promoting the colonization of other bacteria proximal to the epithelial cell layer. Moreover, *R. gnavus* produces sialic acids,^30^ which potentially facilitate the expansion and colonization of other sialic acid-utilizing pathobionts including *E. coli*.^31^ Herein, it is worth noting that colonization with 2-mix weakly but noticeably enhanced the induction of T_H_17 cells, compared to those induced by *E. coli* or *R. gnavus* alone (Figure 3f).

This study has some limitations, including the limited number of subjects assessed and the fact that robust activity in mice may not be physiologically relevant in humans and vice versa. Nevertheless, this study clearly shows T_H_1 cell induction by a CD SI mucosa-associated bacterial strain, thus representing a significant step forward in understanding the interaction between CD SI microbiota and the host immune system. The identified T cell-activating bacteria enriched in the CD mucosa may hence be a promising target for therapeutic intervention.

## Materials and methods

### Patients and sample collection

SI mucosal samples (biopsy or scrape) were harvested from 27 CD patients and 17 non-CD patients who received ongoing clinical care using DBE (**Fig. S1A**). Patients receiving retrograde endoscopy underwent intestinal cleansing procedures from the day before the examination. Midazolam and pethidine were intravenously administered for sedation, and timepidium bromide hydrate or glucagon was used to decrease intestinal peristalsis. Insertion route of DBE (antegrade or retrograde), type of endoscopy (EN-580T, EN-450T5, or EN-450P5, Fujifilm, Tokyo, Japan), and the sampling method were determined on the basis of the patients’ condition. Mucosal biopsies or mucosal scrapes were obtained from the middle SI of each patient, using endoscopic biopsy forceps (Radial Jaw4P, Boston Scientific, MA, USA) or an endoscopic retrieval net (Roth net, US Endoscopy, OH, USA), respectively (**Fig. S1B**). Simultaneously, SI juice (approximately 50 ml) was aspirated through the endoscope before harvesting mucosal specimens. Saliva and stool were sampled one day before the endoscopic examination. For DNA extraction, biopsy samples were suspended in TE10 (10 mM Tris-HCl, 10 mM EDTA) buffer and stored at −80 °C. Mucosal scrape and intestinal juice samples were centrifuged, and the pellets were dissolved in TE10 buffer and stored at −80 °C. For bacterial culturing, biopsy samples were suspended in 20% glycerol/PBS and frozen in liquid nitrogen and then stored at −80 °C. Mucosal scrape samples were centrifuged, and the pellet was dissolved in 20% glycerol/PBS, frozen in liquid nitrogen, and stored at −80 °C.

### DNA extraction and 16S rRNA microbiome sequencing

Frozen samples were thawed and mixed with 800 μl TE10 buffer containing RNase A (final concentration of 100 μg ml^−1^, Invitrogen, MA, USA) and lysozyme (final 15 mg ml^−1^, Sigma, MO, USA). The suspension was incubated for 1 h at 37 °C with gentle mixing. Purified achromopeptidase (Wako, Osaka, Japan) was added at a final concentration of 2,000 units ml^−1^, and the sample was further incubated for 30 min at 37 °C. Thereafter, sodium dodecyl sulfate (final 1%) and proteinase K (final 1 mg ml^−1^, Roche, Basel, Switzerland) were added to the suspension, and the mixture was incubated for 1 h at 55 °C. High-molecular-weight DNA was extracted using phenol:chloroform:isoamyl alcohol (25:24:1), precipitated with isopropanol, washed with 75% ethanol, and resuspended in 50 μl of TE. PCR was performed using 27Fmod 5′-AGRGTTTGATYMTGGCTCAG-3′ and 338R 5′-TGCTGCCTCCCGTAGGAGT-3′ primers targeting the V1–V2 region of 16S rRNA gene. Amplicons generated from each sample (~330 bp) were subsequently purified using AMPure XP (Beckman Coulter, CA, USA). DNA was quantified using a Quant-iT Picogreen dsDNA assay kit (Invitrogen) and a TBS-380 Mini-Fluorometer (Turner Biosystems, CA, USA). The 16S metagenomic sequencing was performed using MiSeq in accordance with the Illumina protocol. Two paired-end reads were merged using the fastq-join program based on overlapping sequences. Reads with an average quality value of <25 and inexact matches to both universal primers were filtered out. Filter-passed reads were used for further analysis after trimming off both primer sequences. For each sample, 3,000 quality filter-passed reads were rearranged in descending order in accordance with their quality value and then clustered into OTUs with a 97% pairwise-identity cutoff using the UCLUST program version 5.2.32 (https://www.drive5.com). The taxonomic assignment of each OTU was carried out on the basis of similarity with the RDP and the National Center for Biotechnology Information (NCBI) genome database using the GLSEARCH program. To determine the microbial prevalence, taxa with a relative abundance >0.1% were considered positive.

### Bacterial culturing and isolation and the generation of gnotobiotic animals

Frozen samples were thawed, serially diluted with PBS, and seeded onto nonselective and selective agar plates (trypticase soy agar for aerobic culturing; EG, BHK, MRS, and CM0619+ SR0107 for anaerobic culturing) (**Fig. S5A**). EG and BHK are nonselective culture media for anaerobic bacteria. MRS is a selective isolation medium for lactobacilli. CM0619 supplemented with SR0107 is a selective isolation medium for non-sporing anaerobes. After culturing under aerobic conditions at 37 °C for 1 d or anaerobic conditions (80% N_2_, 10% H_2_, 10% CO_2_) in an anaerobic chamber (Coy Laboratory Products, MI, USA) at 37 °C for 2–4 d, a total of 572 individual colonies were picked, and their 16S rRNA genes were amplified with universal primers (27Fmod: 5′-AGRGTTTGATYMTGGCTCAG-3′, 1492R: 5′-GGYTACCTTGTTACGACTT-3′) and sequenced. A phylogenetic tree based on these 16S rRNA sequences was constructed using the neighbor-joining method. Individual isolates in the culture collection were grouped as “strains” if their 16S rRNA sequences displayed >98% identity. The resulting strain sequences were compared to those in the NCBI database and to OTUs observed in 16S rRNA analysis to determine closely related species and their corresponding OTUs. To identify isolated bacteria belonging to Enterobacteriaceae, TSI slant (BD, NJ, USA), LIM medium, SIM medium (Eiken Chemical, Tokyo, Japan), and API 20E (BioMérieux, Marcy-l’Étoile, France) were used in accordance with the manufacturers’ instructions. Gram staining was performed using a standard protocol. The CD-associated AIEC strain LF82 used in this study was originally isolated from the ileal mucosa of a CD patient by Darfeuille-Michaud et al.^16^ To generate gnotobiotic mice, each *E. coli* strain, including 35A1, LF82, and MG1655, was aerobically cultured in TS broth at 37°C overnight, and a 200 μl aliquot containing 7–9 × 10^8^ colony-forming units (CFU) was administered to GF mice by oral gavage. *R. gnavus* 131A1 was anaerobically cultured in EG broth or on an EG plate. The colonies on the plate were scraped and resuspended in EG broth. The other seven strains were anaerobically cultured individually in EG or HK broth. Thereafter, for 2-mix or 9-mix, equal volumes of bacterial suspensions were mixed. The mixture of isolates or a single bacterial suspension was orally administered to GF mice, and their colonization was assessed via Gram staining of fecal suspensions. All mice receiving a specified mixture of bacterial strains or a single bacterial suspension maintained in a single gnotobiotic isolator with 12 h/12 h light/dark cycle. GF B6 mice (7–14 weeks old) were used and analyzed 3 weeks after the initial gavage. All animal experiments were approved by the Keio University Institutional Animal Care and Use Committee. All GF and SPF mice were B6 mice obtained from CLEA Japan (Tokyo, Japan).

### Isolation of LP mononuclear cells and flow cytometry

To analyze intestinal lymphocytes, intestines were longitudinally opened and washed with PBS to remove luminal contents. All samples were incubated in 20 ml of Hanks’ balanced salt solution (HBSS) containing 5 mM EDTA for 20 min at 37 °C in a shaking water bath to remove intestinal epithelial cells (IECs). Thereafter, the muscle layer and adipose tissue were manually removed using forceps. The remaining LP layer was cut into small pieces and incubated in 10 ml of RPMI1640 containing 4% fetal bovine serum, 0.5 mg ml^−1^ collagenase D (Roche), 0.5 mg ml^−1^ dispase (Gibco), and 40 µg ml^−1^ DNase I (Roche) for 45 min at 37 °C in a shaking water bath. The digested tissues were washed with 10 ml of HBSS containing 5 mM EDTA, resuspended in 5 ml of 40% Percoll (GE Healthcare, IL, USA), and underlaid with 2.5 ml of 80% Percoll in a 15 ml Falcon tube. Percoll gradient separation was carried out via centrifugation at 900 × g for 30 min at 25 °C. The fraction containing lymphocytes was harvested from the interface of the two layers and washed with RPMI1640 containing 10% FBS. For cytokine detection, the cells were stimulated with 50 ng ml^−1^ PMA and 750 ng ml^−1^ ionomycin (both from Sigma) in the presence of GolgiStop (BD) at 37 °C for 3.5 h. After labeling with Ghost Dye 780 (Tonbo Biosciences, CA, USA), the cells were permeabilized and stained with anti-TCRβ (BV605; Biolegend, CA, USA), anti-CD4 (BV510; Biolegend), anti-TCRγδ (BV421; Biolegend), anti-IFN-γ (FITC; Biolegend), anti-IL-17A (PerCP-Cy5.5; eBioscience, CA, USA), anti-TNF-α (PE/Cy7; Biolegend), anti-RORγt (APC; eBioscience), and anti-DR3 (PE; Biolegend) antibodies, using the Foxp3/Transcription Factor Staining Buffer Kit (eBioscience) in accordance with the manufacturer’s instructions. All data were collected using a BD FACS Aria IIIu instrument and analyzed with Flowjo software (TreeStar). CD4 T cells were defined as the CD4^+^ TCRβ^+^ population within the live-cell gate.

### Anti-IL10 R antibody-mediated colitis model

The anti-IL10 R antibody-mediated colitis mouse model was generated as described by Schiering et al.^17^ Briefly, GF wild-type B6 mice were monocolonized with the *E. coli* 35A1, LF82, or MG1655 strain on day 1, followed by weekly intraperitoneal injections with anti-mouse IL10 R antibody (1 mg/body) (Bio X Cell, NH, USA) from day 1 until the end of the experiment. The mice were analyzed 5 weeks after the initial colonization.

### Histological analysis

To evaluate the development and severity of intestinal inflammation, the cecum and proximal and distal colon were fixed with 4% paraformaldehyde, embedded in paraffin, sectioned, and stained with hematoxylin and eosin. The degree of inflammation was blindly graded according to the following criteria: inflammatory cell infiltration (score, 0–4), mucosa thickening (score, 0–4), goblet cell depletion (score, 0–4), crypt abscess (score, 0–4), and destruction of architecture (score, 0–4). The final histological score was defined as the sum of the scores of these parameters.

### RNA isolation and qPCR

Total RNA was isolated from scraped colonic epithelium using TRIzol reagent (Invitrogen) per the manufacturer’s instructions. cDNA was synthesized using ReverTra Ace qPCR RT Master Mix (TOYOBO, Osaka, Japan), and qPCR was performed using Thunderbird SYBR qPCR Mix (TOYOBO) on a LightCycler 480 (Roche). The following primer pairs were used: *Actb*, 5ʹ-TATGCCAACACAGTGCTGTC-3ʹ and 5ʹ-ACCGATCCACACAGAGTACTTG-3ʹ; *Tnfa*, 5ʹ-TCATACCAGGAGAAAGTCAACCTC-3ʹ and 5ʹ-GTATATGGGCTCATACCAGGGTTT-3ʹ.

### Statistical analysis

Statistical analysis was performed using GraphPad Prism 7 software (GraphPad Software, CA, USA). In particular, the two-tailed unpaired Student’s *t*-test (parametric) was used for all comparisons between two groups. One-way analysis of variance (ANOVA) followed by Tukey’s post hoc test (parametric) was used for multiple-group comparisons. A chi-square test was used to compare the microbial prevalence between different groups. Two-way ANOVA followed by Bonferroni’s post hoc test was used for data with two grouping variables. Bray-Curtis distance-based non-metric multidimensional scaling (NMDS) and permutational multivariate analysis of variance (PERMANOVA) with pairwise multilevel comparison were carried out using R software (version 3.6.1, package vegan and phyloseq). For multiple comparisons between two groups, multiple t-tests with the FDR approach and LEfSe were used.^32^ A *P* value < .05 was considered statistically significant.

## Supplementary Material

Supplemental Material
