# Supplementary material for: TH1 cell-inducing Escherichia coli strain identified from the small intestinal mucosa of patients with Crohn’s disease
Source: Gut Microbes. 2020 Jul 20;12(1):1788898. doi: 10.1080/19490976.2020.1788898 (PMC7524366; doi:10.1080/19490976.2020.1788898)
Supplement: Supplemental Material [file KGMI_A_1788898_SM3562.pdf]

## Supplemental Material

### Figure S1

**A**

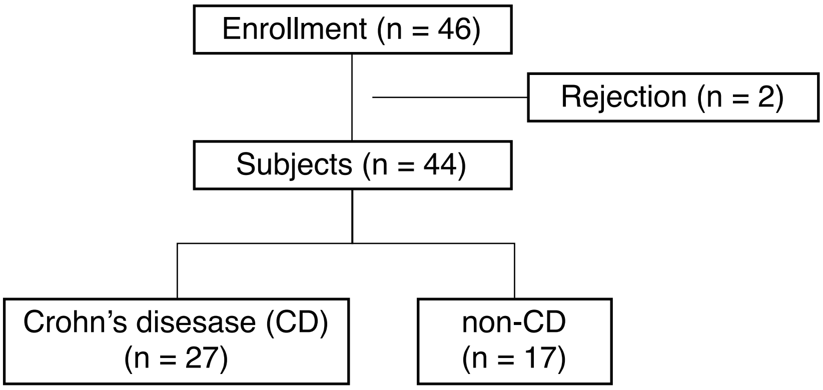


**B**

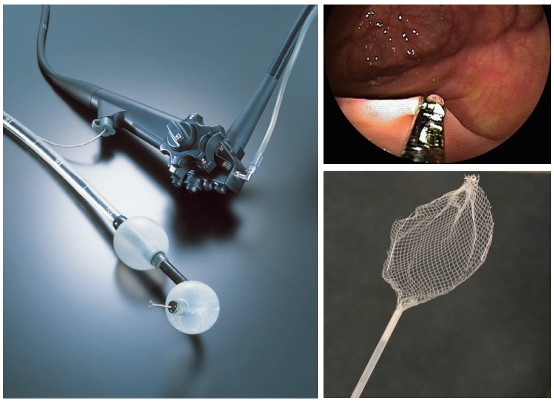

**Figure S1. Participants enrolled in this study and equipment used for sample collection.** (A) A schematic representation of patient enrollment. (B) Images of the double-balloon enteroscope, mucosal biopsy, and Roth net.

## Figure S2

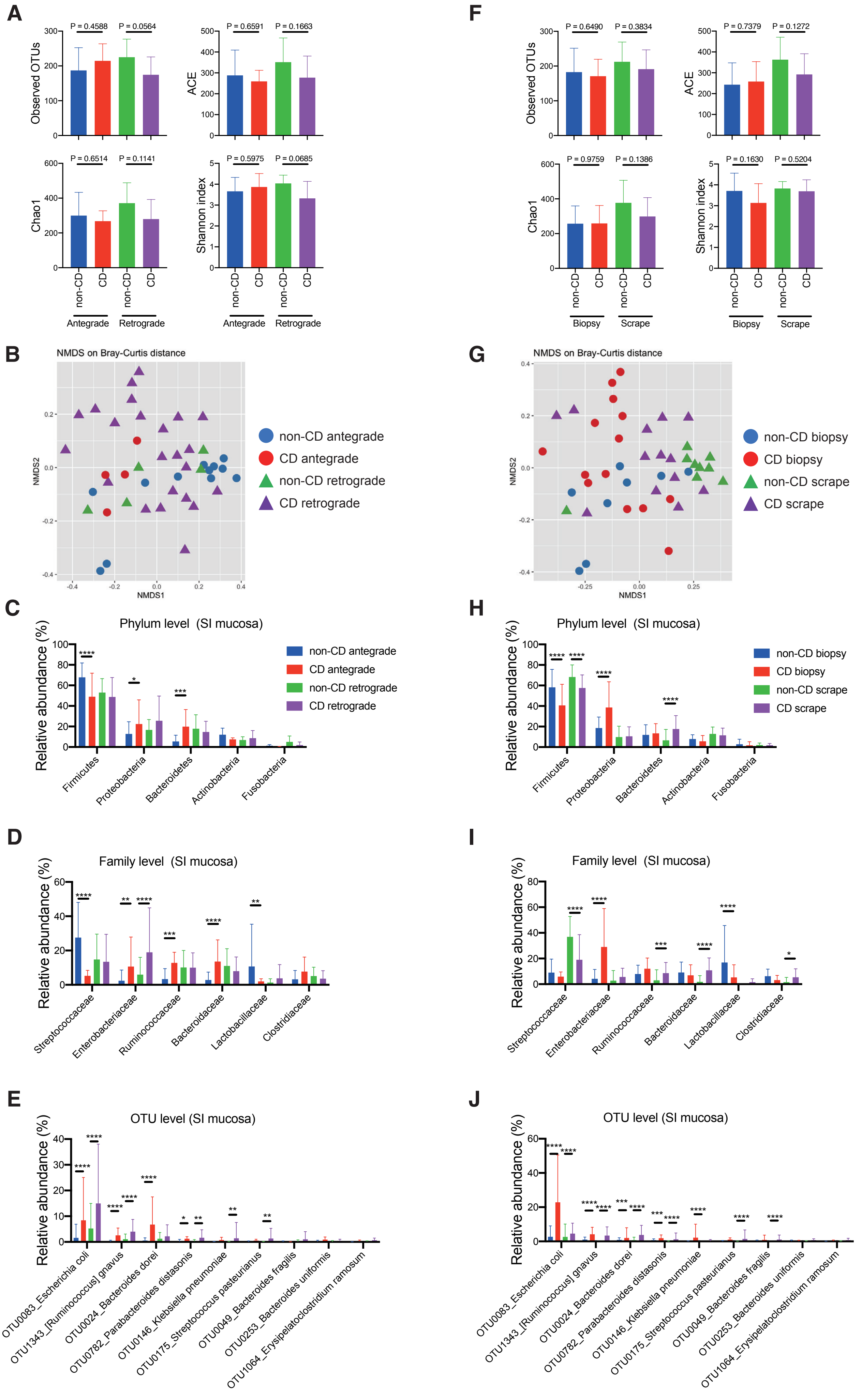

A
P = 0.4588
P = 0.0564
Observed OTUs
P = 0.6591
P = 0.1663
ACE
P = 0.6514
P = 0.1141
Chao1
P = 0.5975
P = 0.0685
Shannon index
non-CD
CD
Antegrade
Retrograde
F
P = 0.6490
P = 0.3834
P = 0.7379
P = 0.1272
P = 0.9759
P = 0.1386
P = 0.1630
P = 0.5204
Biopsy
Scrape
B
NMDS on Bray-Curtis distance
NMDS1
NMDS2
non-CD antegrade
CD antegrade
non-CD retrograde
CD retrograde
G
NMDS on Bray-Curtis distance
non-CD biopsy
CD biopsy
non-CD scrape
CD scrape
C
Phylum level (SI mucosa)
Relative abundance (%)
Firmicutes
Proteobacteria
Bacteroidetes
Actinobacteria
Fusobacteria
H
Phylum level (SI mucosa)
D
Family level (SI mucosa)
Streptococcaceae
Enterobacteriaceae
Ruminococcaceae
Bacteroidaceae
Lactobacillaceae
Clostridiaceae
I
Family level (SI mucosa)
E
OTU level (SI mucosa)
OTU0083_Escherichia coli
OTU1343_[Ruminococcus] gnavus
OTU0024_Bacteroides dorei
OTU0782_Parabacteroides distasonis
OTU0146_Klebsiella pneumoniae
OTU0175_Streptococcus pasteurianus
OTU0049_Bacteroides fragilis
OTU0253_Bacteroides uniformis
OTU1064_Erysipelatoclostridium ramosum
J
OTU level (SI mucosa)

**Figure S2. Microbiota structure in the small intestinal (SI) mucosa of Crohn’ s disease (CD) patients based on insertion route and sampling method.** (A-E) Analyses using SI mucosal samples from antegrade or retrograde insertion only. (F-J) Analyses using SI biopsies or scraped specimens only. Alpha-diversity indices (A, F); Bray-Curtis distance-based non-metric multidimensional scaling (NMDS) plot (B, G); and relative abundance of bacterial taxa at the phylum (C, H), family (D, I), and OTU level (E, J) of the SI mucosal microbiota. Each bar in (A, C-F, H-J) represents the mean values of groups. Each point in (B, G) represents an individual sample. Error bar, SD. P or FDR value * < 0.05; ** < 0.01; *** < 0.001; **** < 0.0001, unpaired Student’ s t-test (A, F), multiple t-tests with false discovery rate (FDR) approach (C-E, H-J).

# Figure S3

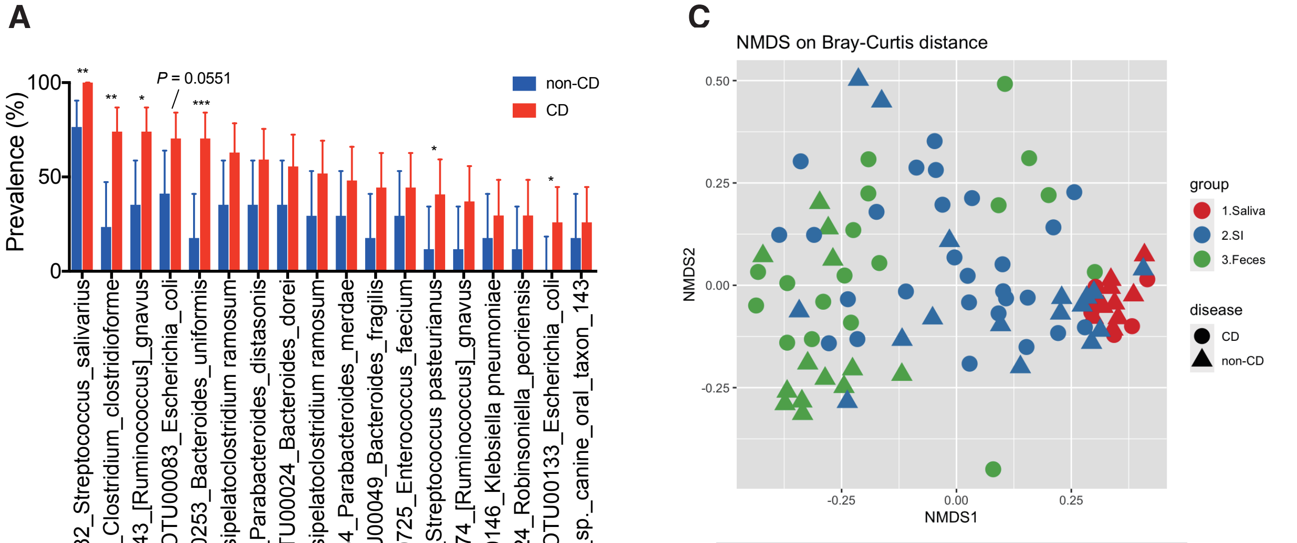


| Pairs | $R^2$ | Adjusted *P* value |
|---|---|---|
| **SI vs. Saliva** | 0.2737909 | 0.003 |
| **SI vs. Feces** | 0.1433416 | 0.003 |
| **Saliva vs. Feces** | 0.6879742 | 0.003 |

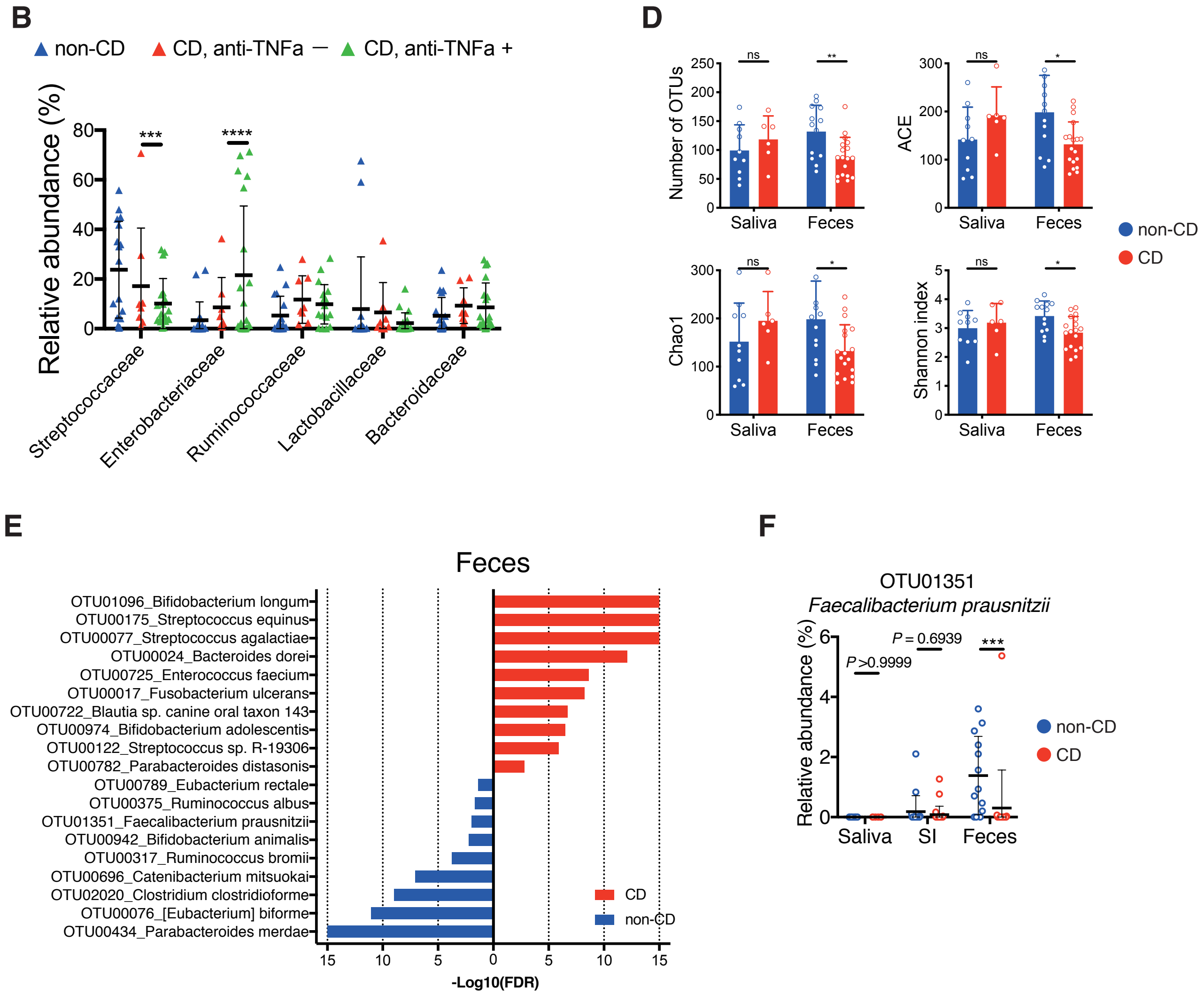

**Figure S3. Differences between the small intestinal (SI), salivary, and fecal microbiota.** (A) A histogram of the prevalence of 18 CD-enriched bacteria. The prevalence was calculated by dividing the number of subjects whose SI mucosal samples contained the indicated bacteria with a relative abundance >0.1% by the total number of subjects. (B) Relative abundance of bacterial taxa in the SI mucosal microbiota at the family level. Statistical comparison between CD patients treated with anti-TNF-α antibody therapy (green triangles) and the other CD patients (red triangles). (C) Bray-Curtis distance-based non-metric multidimensional scaling (NMDS) plot of the SI mucosa, saliva, and feces (upper). Permutational multivariate analysis of variance (PERMANOVA) statistics among anatomical locations (lower). (D) Alpha-diversity indices of the microbiota of saliva and feces. (E) A histogram of the false discovery rate (FDR) calculated by comparing fecal samples between CD and non-CD controls. Only significant taxa (FDR < 0.05) are shown. (F) Relative abundance of *F. prausnitzii* in saliva, SI mucosa, and feces. Each bar in (D) and thick bar in (B, F) represent the mean of a group. Each point in (B-D, F) represents an individual sample. Error bar, 95% confidence interval (A), SD (B, D, F). P or FDR value * < 0.05; ** < 0.01; *** < 0.001; **** < 0.0001; ns, not significant (P or FDR > 0.05), chi-square test (A), multiple t-tests with FDR approach (B, E), unpaired Student' s t-test in (D, F).

# Figure S4

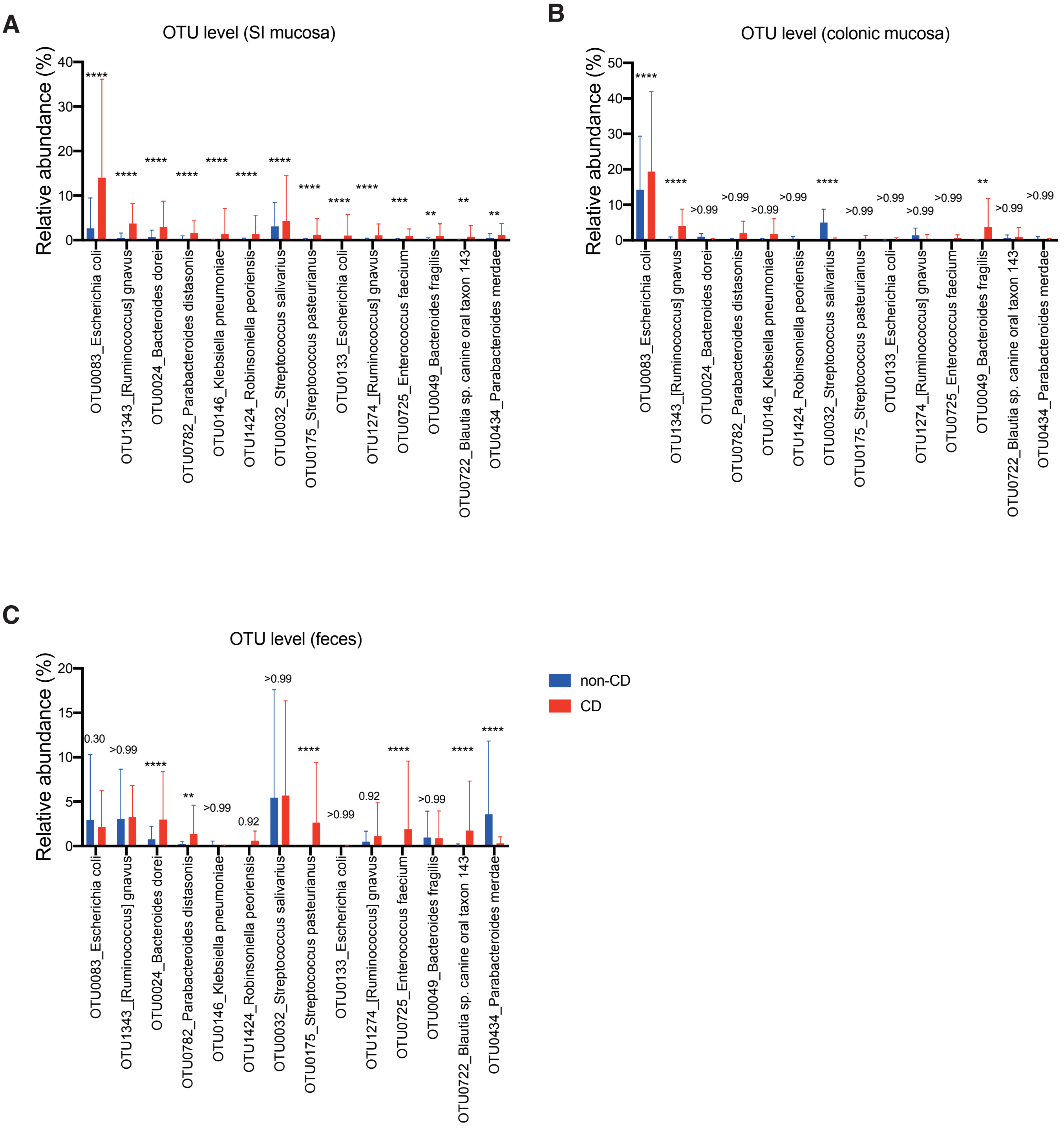


**Figure S4. Taxa that were enriched in the small intestinal (SI) mucosa but not the feces of Crohn' s disease (CD) patients.** (A-C) The relative abundance of CD-associated taxa in the SI mucosa (A), colonic mucosa (B), and feces (C). Only 14 OTUs significantly enriched in the SI mucosa of CD patients are shown. Each bar represents the mean of a group. Error bar, SD. *FDR < 0.05; **FDR < 0.01; ***FDR < 0.001; ****FDR < 0.0001, multiple t-tests with FDR approach.

## Figure S5

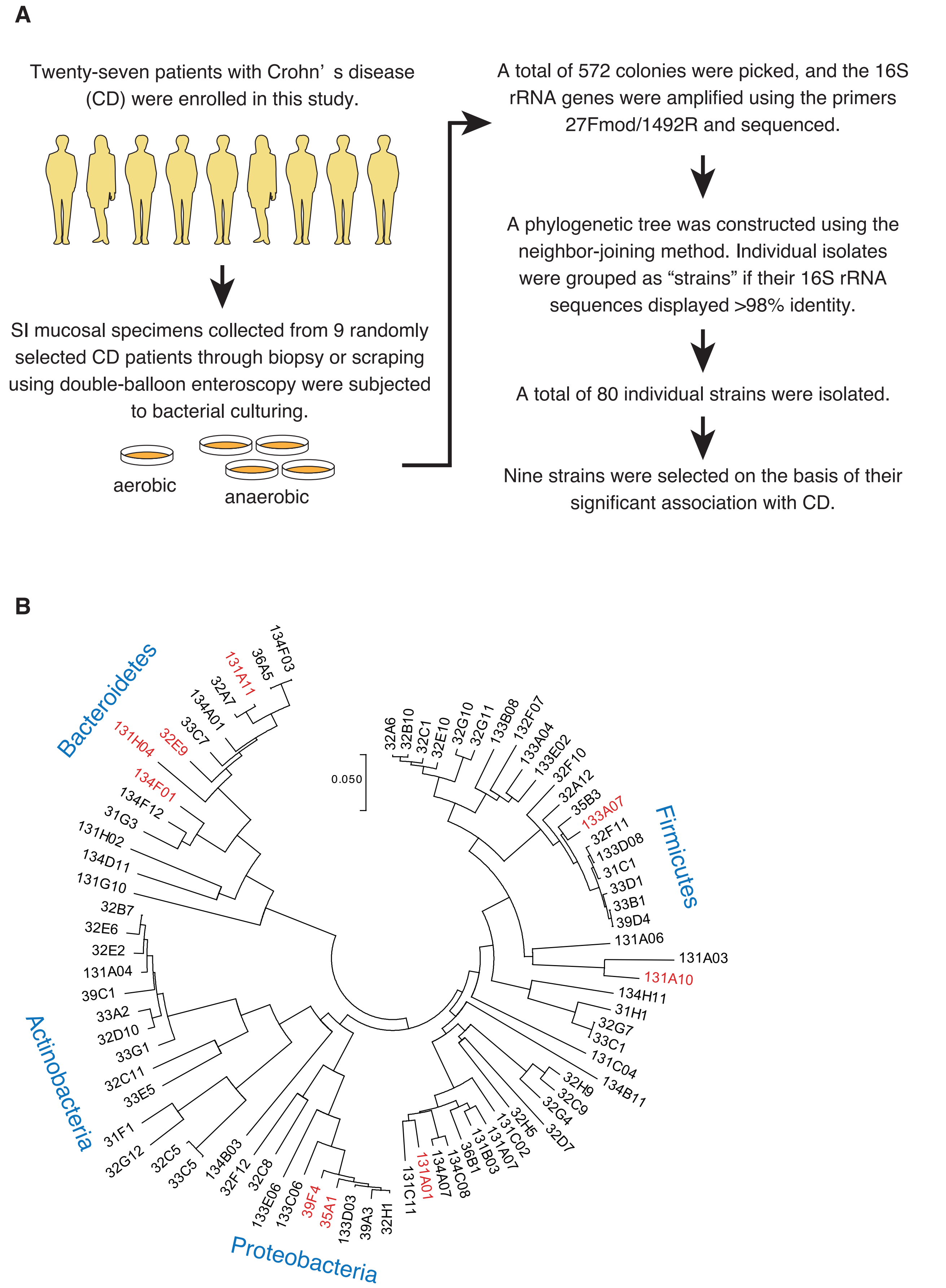


**Figure S5. Schematic representation of bacterial isolation from the small intestinal (SI) samples of Crohn’ s disease (CD) patients and the phylogenetic tree of 80 isolated bacteria.** (A) Schematic representation of bacterial isolation from CD SI samples. (B) The phylogenetic tree generated from analysis based on 16S rRNA sequences of 80 strains isolated from CD SI samples. The 9 strains used in gnotobiotic experiments (9-mix, see Fig. 2G and 3A-D) are marked in red.

**Figure S6**

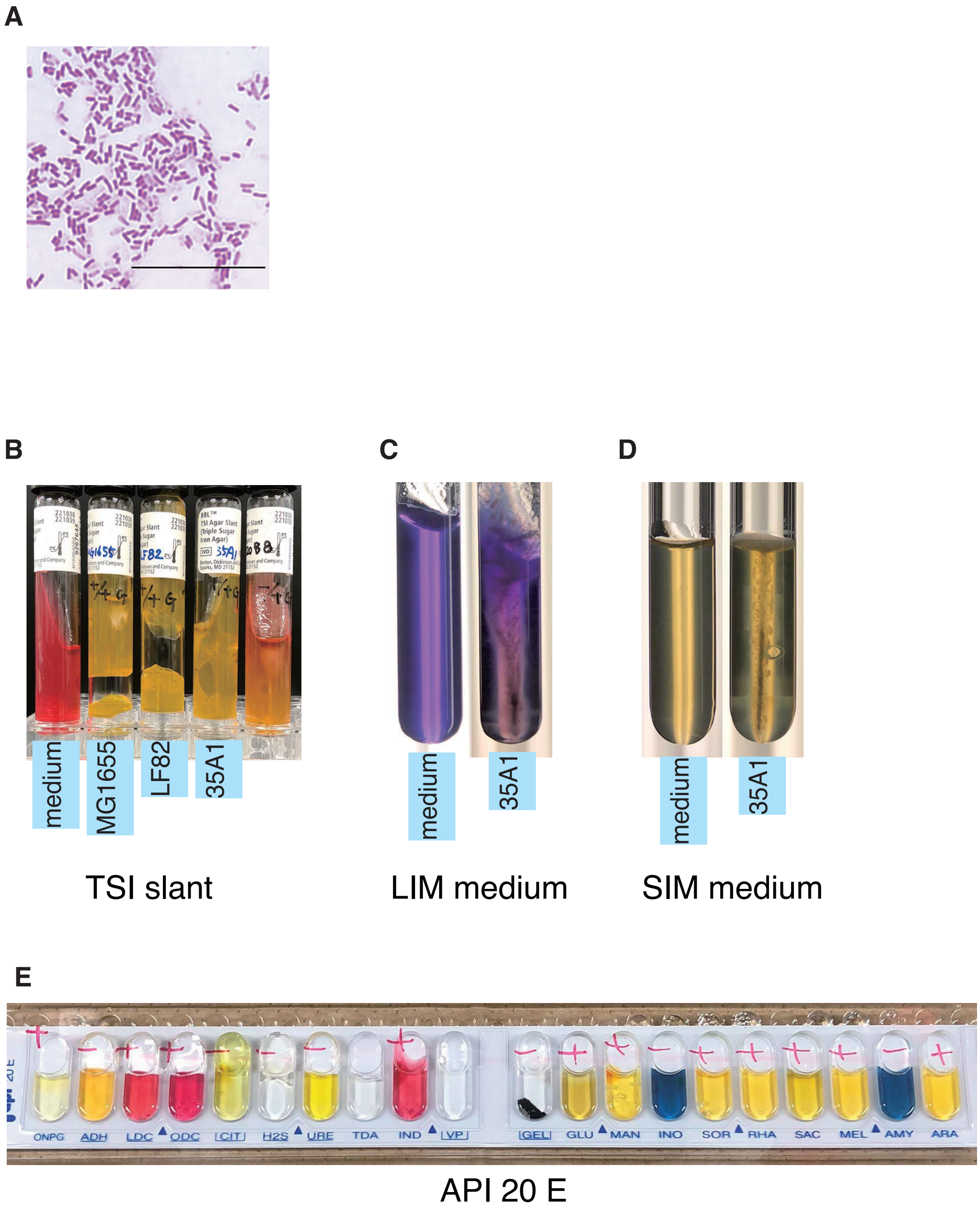


**Figure S6. Identification of the *Escherichia coli* 35A1 strain isolated from the small intestinal (SI) samples of Crohn' s disease (CD) patients.** (A) Representative image of Gram staining of in vitro-cultured *E. coli* 35A1. (B-E) Biochemical tests (TSI slant, LIM/SIM medium, and API 20 E test strip) revealed that the strain was positive for sugar fermentation, motility, and indole production. Scale bar = 20 µm (A).

**Figure S7**

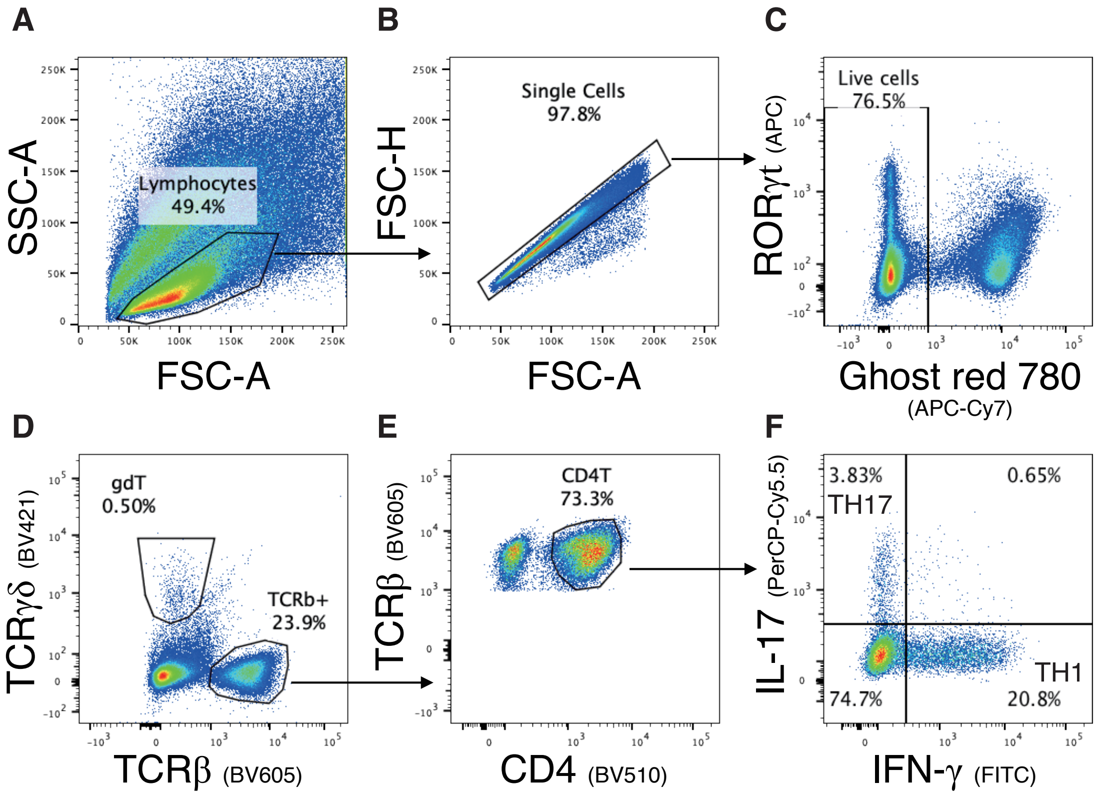


**Figure S7. Gating strategy for flow cytometry analysis.** (A, B) The intestinal lamina propria lymphocytes were gated to eliminate debris, and single cells were gated. (C) The living cells were gated by Ghost red 780. (D, E) The TCRβ-positive cells and the CD4-positive cells were gated. (F) CD4 T cells were defined as the CD4+ TCRβ+ population within the live-cell gate.

## Figure S8

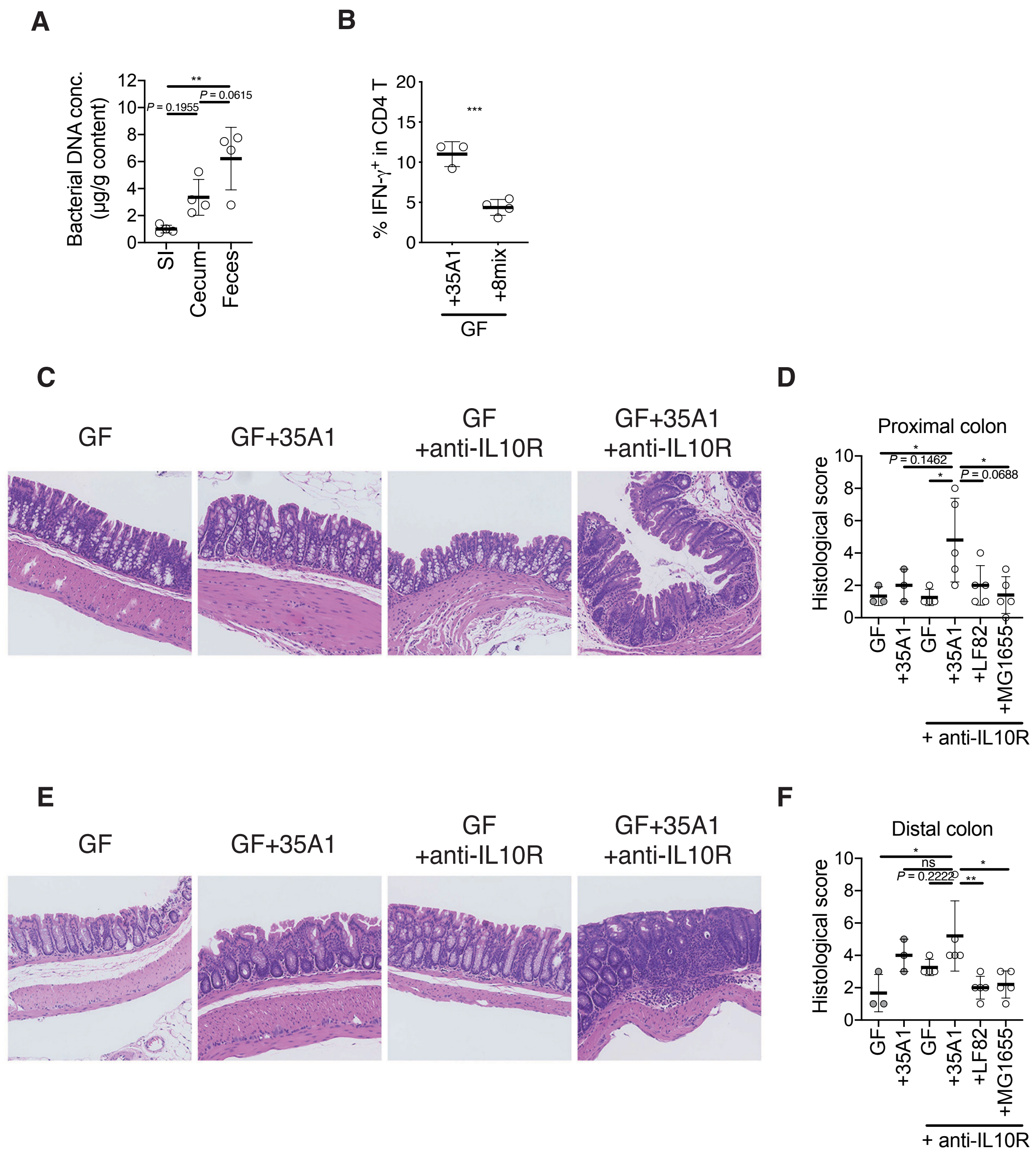


**Figure S8. Strain-dependent induction of intestinal inflammation by Crohn' s disease (CD)-associated *Esherichia coli*.** (A) Bacterial DNA was extracted from fecal pellets and luminal contents of the cecum and SI of *E. coli* 35A1-monocolonized mice. Bacterial DNA concentration was determined by qPCR. (B) Percentage of IFN-γ+ cells among colonic lamina propria (LP) CD4 T cells. Germ-free (GF) C57BL/6 (B6) mice were orally inoculated with 35A1 alone or 8-mix (9-mix without *E. coli* 35A1). (C-F) Representative hematoxylin and eosin staining (C, E) and histological colitis scores (D, F) of the proximal (C, D) and distal colon (E, F) of *E. coli* 35A1, LF82, or MG1655-monocolonized colitis model mice. Each strain was orally administered to GF wild-type B6 mice on day 1, followed by weekly intraperitoneal injections with anti-mouse IL10 receptor antibody (1 mg/body) from day 1 until the end of the experiment. Each point in (A, B, D, F) represents an individual mouse. Each thick bar in (A, B, D, F) represents the mean of a group. Error bar, SD. *P < 0.05; **P < 0.01; ***P < 0.001, one-way ANOVA with Tukey's post hoc test (A, D, F), unpaired Student' s t-test (B).

**Table S1**

| OTU | Closest species | %Id | Strain ID (9mix) | Abundance (%) | | | Prevalence (%) | | |
|---|---|---|---|---|---|---|---|---|---|
| | | | | non -CD | CD | Fold change | non -CD | CD | Fold change |
| OTU00024 | *Bacteroides dorei* | 99.68 | 131H4 | 0.69 | 2.88 | 4.1 | 35.3 | 55.6 | 1.6 |
| OTU00049 | *Bacteroides fragilis* | 97.77 | 32E9 | 0.15 | 0.90 | 6.2 | 17.6 | 44.4 | 2.5 |
| OTU00253 | *Bacteroides uniformis* | 99.68 | 131A11 | 0.18 | 0.48 | 2.6 | 17.6 | 70.4 | 4.0 |
| OTU00722 | *Blautia sp. canine oral taxon 143* | 99.69 | | 0.07 | 0.80 | 11.7 | 17.6 | 25.9 | 1.5 |
| OTU00682 | *Clostridium clostridioforme* | 99.69 | | 0.15 | 0.71 | 4.7 | 23.5 | 74.1 | 3.1 |
| OTU00725 | *Enterococcus faecium* | 98.78 | | 0.14 | 0.94 | 6.8 | 29.4 | 44.4 | 1.5 |
| OTU01064 | *Erysipelatoclostridium ramosum* | 99.77 | 131A10 | 0.10 | 0.53 | 5.5 | 35.3 | 63.0 | 1.8 |
| OTU00239 | *Erysipelatoclostridium ramosum* | 98.68 | | 0.05 | 0.26 | 5.6 | 29.4 | 51.9 | 1.8 |
| OTU00083 | *Escherichia coli* | 99.23 | 35A1 | 2.66 | 14.02 | 5.3 | 41.2 | 70.4 | 1.7 |
| OTU00133 | *Escherichia coli* | 98.71 | | 0.00 | 1.03 | 525.7 | 0 | 25.9 | - |
| OTU00146 | *Klebsiella pneumoniae* | 99.63 | 39F4 | 0.05 | 1.31 | 26.6 | 17.6 | 29.6 | 1.7 |
| OTU00782 | *Parabacteroides distasonis* | 100 | 134F1 | 0.29 | 1.58 | 5.5 | 35.3 | 59.3 | 1.7 |
| OTU00434 | *Parabacteroides merdae* | 100 | | 0.47 | 1.16 | 2.5 | 29.4 | 48.1 | 1.6 |
| OTU01424 | *Robinsoniella peoriensis* | 94.44 | | 0.13 | 1.35 | 10.4 | 11.8 | 29.6 | 2.5 |
| OTU01343 | *[Ruminococcus] gnavus* | 99.65 | 131A1 | 0.45 | 3.78 | 8.4 | 35.3 | 74.1 | 2.1 |
| OTU01274 | *[Ruminococcus] gnavus* | 98.15 | | 0.10 | 1.10 | 10.8 | 11.8 | 37.0 | 3.1 |
| OTU00175 | *Streptococcus pasteurianus* | 100 | 133A7 | 0.08 | 1.23 | 14.9 | 11.8 | 40.7 | 3.5 |
| OTU00032 | *Streptococcus salivarius* | 99.68 | | 3.11 | 4.31 | 1.4 | 76.5 | 100.0 | 1.3 |

**Table S1. List of 18 enriched bacteria in Crohn’ s disease (CD) patients.** The closest species or strain, percentage similarity (%) in the National Center for Biotechnology Information (NCBI) genome database, strain IDs of bacteria in the 9-mix, mean abundance, and prevalence of each group are indicated. The prevalence was calculated by dividing the number of subjects whose SI mucosal samples contained the indicated bacteria with a relative abundance >0.1% by the total number of subjects.
